# Social Media Use in Adolescents With Functional Abdominal Pain

**DOI:** 10.3389/fped.2020.592972

**Published:** 2020-11-24

**Authors:** Eshan Samuel, Sharmistha Lahiri, Syed Hashmi, Fernando Navarro

**Affiliations:** Divisions of Gastroenterology and Pediatric Research Center, Department of Pediatrics, University of Texas Health McGovern Medical School and the Children's Memorial Hermann Hospital, Houston, TX, United States

**Keywords:** social media, anxiety, depression, abdominal pain, pediatric

## Abstract

Social media use is increasing in children in the U.S., which could be related to the high prevalence of functional gastrointestinal disorders in this population.

**Objective:** To investigate the relationship of social media use with the severity of gastrointestinal symptoms in patients with a functional abdominal pain or irritable bowel syndrome diagnosis.

**Study Design:** We administered a questionnaire to collect information about screen time, demographics, and a modified Validated Varni PedsQL Gastrointestinal symptom scale which assesses the severity of gastrointestinal (GI) symptoms.

**Results:** We surveyed a total of 59 subjects, which included 26 subjects with functional abdominal pain and 33 age-matched healthy controls. The median score across all 8 scales was about a third less for cases (median: 63; IQR: 55–78) than controls (median: 93; IQR: 83–95) (*p* < 0.001). Mean screen time in the study group (341 min/day) was very similar to that in the control group (331 min/day). There was no statistically significant association between screen time per day and the number of platforms used for either the cases or controls. YouTube (92%) and Instagram (88%) were the first and second most popular platforms used by the children with functional abdominal pain; Instagram (97%) and Snapchat (82%) were the most popular platforms in the healthy controls. Interestingly, social media were more often used for entertainment, reading, and productivity by the children with functional abdominal pain (*p* < 0.05).

**Conclusion:** The amount of screen time/day and the number of social media platforms used does not correlate with the severity of abdominal pain and other GI symptoms in adolescents with FGID. Further research will be needed to confirm if the platforms are used differently by children with functional gastrointestinal disorder.

## Introduction

Social media use has been receiving a substantial amount of attention over the past several years, as the popularity of social networking communities continues to grow. There are currently a plethora of social media networking websites and applications. A few of the most popular platforms include Facebook, Instagram, Snapchat, Twitter, Linked-In, YouTube, WhatsApp, and Pinterest. These platforms have become a primary modality of communication for many growing adolescents, as they provide a fast, inexpensive and convenient method of communication. However, this relatively new phenomenon has been of particular interest to the medical community. Not only the time spent in social media but also the use of multiple social media platforms has been linked to psychological disorders such as anxiety and depression (1–3).

Patients with functional gastrointestinal disorders (FGID) are diagnosed based on published ROME criteria given their clinical symptoms in the absence of inflammatory, metabolic, or anatomic abnormalities. In our experience many of our patients with functional gastrointestinal disorders often complain of psychological symptoms. In a systematic review of childhood recurrent abdominal pain (RAP) in western countries, the prevalence was reported to range between 0.3 and 19% (4). Children with RAP have been reported to have psychological comorbidities such as anxiety, traumatic life events, stress and depression (3).

We speculated that the vast majority of our patients with FGID such as functional abdominal pain and irritable bowel syndrome (IBS) use social media. Given the association between social media use and mood disorders in adolescents, we hypothesized that the amount of time dedicated to social media was correlated with the severity of their gastrointestinal symptoms.

According to a report by S.O'Dea, currently there are more than 100 million iPhone users, which accounts for about 45 percent of total mobile phone users in the United States, and this number keeps increasing (5).

## Aims

### Primary Aim

To quantify social media use in adolescents between 13 and 18 years of age with FGID which includes functional abdominal pain and IBS and healthy aged-matched controls, followed at the UT Pediatric Gastroenterology Clinic via a prospective study.

### Secondary Aims

#### Secondary Aim 1

To determine the correlation between the number of social media platforms used by children with FGID in the last year, and the severity of their symptoms.

#### Secondary Aim 2

To determine the correlation between the time spent on social media by the patients with FGID in the last year and the severity of the symptoms.

## Methods

### Inclusion Criteria

Patients aged 13–18 years of age diagnosed with FGID, who use an iPhone, were enrolled. We included subjects aged 13–18 years, as children below the age of 13 years old are not legally permitted to open an account on social media platforms. Apple iPhone has a unique feature through which we are able to easily track the screen time, so we decided to focus our survey study on adolescents who own an iPhone. This feature gave us accurate quantifiable and standardized data without any recall bias.

### Exclusion Criteria

We excluded patients with a diagnosis of organic conditions, including inflammatory bowel disease, celiac disease, *H. pylori* infection, pancreatitis, cholecystitis, cholelithiasis, urolithiasis, or pelvic inflammatory disease. Those using other mobile phone types (e.g., android devices) were excluded (as they are unable to numerically track social media usage in a similar manner).

We received approval from (HSC-MS-19-0395) the University of Texas Institutional Review Board (IRB) before starting the research. Research took place between September 2019 and April 2020. We administered the questionnaire to 63 subjects. However, among the 63 of patients we invited to participate in the study, four patients were not able to take part because they have Android devices, so our final sample size was 59 patients.

The survey included a voluntary assent form, which the subjects read before agreeing to participate.

We surveyed 26 subjects with a known diagnosis of FGID, followed at the pediatric gastroenterology clinic and 33 age-matched healthy controls followed at the general pediatric clinic for well-child visits, who otherwise had no significant medical problems. Among the 26 subjects in the FGID group, seven had the diagnosis of functional abdominal pain, 19 had the diagnosis of IBS. Functional abdominal pain patients were diagnosed after ruling out the potential organic conditions which can explain the patient symptoms and IBS patients were diagnosed using ROME 4 criteria (6). We administered the questionnaires both in person at the clinic during their regular appointments and via phone using a pre-defined telephone template. On the phone we administered the questionnaire by asking each of the questions listed and completing the answers as reported by the subjects.

This questionnaire included information about demographics including gender, age, academic grades, type of operating system on their phone (IOS or. Android), social media platform(s) used in the last year, and screen time recorded on their iPhone (including time spent on subcategories of social networking, reading and reference, entertainment, productivity, and weekly total). We also recorded medications used in the last year.

### Peds QL Gastrointestinal Symptoms Scale

We modified the Varni Peds QL Gastrointestinal Symptoms Scale (4) and which included eight components instead of ten. We included: Abdominal Pain Scale (six items), Abdominal Discomfort When Eating Scale (five items), Trouble Swallowing /Dysphagia Scale (three items), Heartburn and Reflux Scale (four items), Nausea and Vomiting Scale (four items), Gas and Bloating Scale (seven items), Constipation Scale (14 items), and Diarrhea Scale (seven items). We excluded food and drink limits scale (6 items) and “blood in the poop” scale (two items), as they were not indicated in these otherwise healthy children. We used the scoring methods, as described by Varni et al. (4). We used child self-report form specific for the age 13–18 years, which asked how much of the particular problem a subject had in the past 1 month. This scoring system used a 5-point response scale, which included 0 = never a problem, 1 = almost never a problem, 2 = sometimes a problem, 3 = often a problem, 4 = almost always a problem. After the self-reporting of the symptoms on the questionnaire by the subject, items on the modified Varni QOL were reverse- scored and linearly transformed to a 0–100 scale (0 = 100, 1 = 75, 2 = 50, 3 = 25, 4 = 0). For the final QL Gastrointestinal Symptom score (50 Items), the mean was calculated as the sum of the items divided by the number of items in the eight modified PEDS QL Gastrointestinal symptoms scale (7). Lower scores demonstrate worse GI-related quality of life, and higher scores are indicative of a better GI-related quality of life.

### Statistical Analysis

The categorical variables describing the number of study subjects that reported using specific social media platforms or specific medications are presented as frequencies (with percentages). Continuous variables are described as medians (with interquartile ranges). Comparisons of the categorical and continuous variables across case/control categories was performed using Fisher exact test or Mann-Whitney ranked sum test, respectively. Spearman correlation coefficients were utilized to assess the monotonic relationship between continuous variables. Multivariable linear regression models were used to assess screen time for both QL scores and case-control status while adjusting for each other. Statistical significance was assumed at a Type 1 error rate of 5%. All analysis was performed using Stata (v14. Stata Corp., College Station, Texas) and GraphPad Prism (v 6.0 for Mac OS X. GraphPad Software, La Jolla, CA).

## Results

Altogether, 26 cases, including nine cases with functional abdominal pain, 19 cases with irritable bowel syndrome and 33 control subjects were recruited for the study. The median age was 16 years (IQR: 15–17) in both groups (*p* = 0.607). Both groups have female predominance, with 88.46% females in the study group vs. 72.72% in the control group (Table 1). We have 13 males and 46 females who participated in the study. There was a difference in racial/ethnic distribution between the two groups, with a greater proportion of non-Hispanic Caucasians (*n* = 11, 42%) and Hispanics (*n* = 10, 38%) in the FGID group and a greater proportion of and Hispanics (*n* = 15, 45%) and African Americans (*n* = 11, 33%) in the control group (*p* = 0.001).

**Table 1:** Demographics.

**Factor**	**Total sample**	**FGID group (%)**	**Control group (%)**
Gender			
N	59	26	33
Male	20	3 (12)	9 (27)
Female	80	23 (88)	24 (73)
Race			
Hispanic	25	10 (38)	15 (45)
Asian	5	1 (4)	4 (12)
Caucasian	14	11 (42)	3 (9)
African American	12	1 (4)	11 (33)
Others	3	3 (12)	0 (0)
Age (average age in years)			
	15.8	16.07	15.8
Education (academic grade average)			
	10th	10th	11th

*Both groups had a female predominance, with 88% of females in the FGID group vs. 73% in the control group. A majority of the subjects included in the study and control groups were Hispanic (42%). Mean age in the FGID group was 16.0 years vs. 15.8 years in the control group. FGID, Functional Gastrointestinal Disorder*.

Correlations between screen time and PEDS QL scores for GI symptoms among case subjects were none to poor (range −0.29 to 0.18, *p* > 0.05 for all). There was no significant difference between PEDS QL scores for GI symptoms of functional abdominal pain and Irritable bowel syndrome. On Pearson Correlation analysis, we did not find any linear association between screen time and gastrointestinal symptoms including diarrhea, constipation, bloating, heartburn and reflux, nausea and vomiting (Supplementary Graph 1 in Supplementary Material). However, when the correlations were compared within controls, there was moderate correlations between screen time and nausea/vomiting (rho = 0.37, *p* = 0.035) and screen time and constipation (rho = 0.36, *p* = 0.038). Additionally, the correlations for nausea/vomiting, constipation and diarrhea were significantly different between cases and controls (*p* = 0.013, *p* = 0.019, and *p* = 0.047), respectively.

There was no statistically significant association between screen time per day and the number of platforms used for either the cases or controls (Table 2). This lack of an association was present even after number of platforms used were restructured into few (1 or 2), medium (3 or 4) or high (5 or 6) categories. There was also no difference in screen time identified in multivariable models where case status and number of platforms used were adjusted for each other (regression model individual *p* > 0.05 for all).

**Table 2:** Social media use for patients with functional GI disorders and healthy controls.

	**Cases, *n* = 26**	**Controls, *n* = 33**	***p*-value**
**Specific platform used**, ***n*** **(%)**			
Facebook	12 (46)	11 (33)	0.421
Instagram	23 (88)	32 (97)	0.311
LinkedIn	0 (0)	0 (0)	-
YouTube	24 (92)	25 (76)	0.161
WhatsApp	3 (12)	3 (9)	1.000
Snapchat	22 (85)	27 (82)	1.000
Pinterest	8 (31)	7 (21)	0.549
Twitter	9 (35)	18 (55)	0.188
Tumblr	1 (4)	0 (0)	0.441
Netflix	2 (8)	0 (0)	0.190
TikTok	2 (8)	0 (0)	0.190
Discord	1 (4)	0 (0)	0.441
Vsco	1 (4)	0 (0)	0.441
Number of platforms used, *n* (%)	4 (3–5)	4 (3–5)	0.772
Time spent minutes, median (IQR)			
Screen time/day	309 (219–375)	297 (206–449)	0.867
Social networking	362 (151–720)	211 (106–410)	0.225^*^
Reading and reference	43 (28–120)	0 (0–35)	0.001^*^
Entertainment/day	230 (61–449)	49 (4–258)	0.046^*^
Productivity	81 (42–220)	0 (0–36)	0.002^*^
Weekly total	954 (429–2470)	860 (639–2023)	0.901

*IQR = interquartile range*.

* *Statistically significant*.

**Figure 1:**
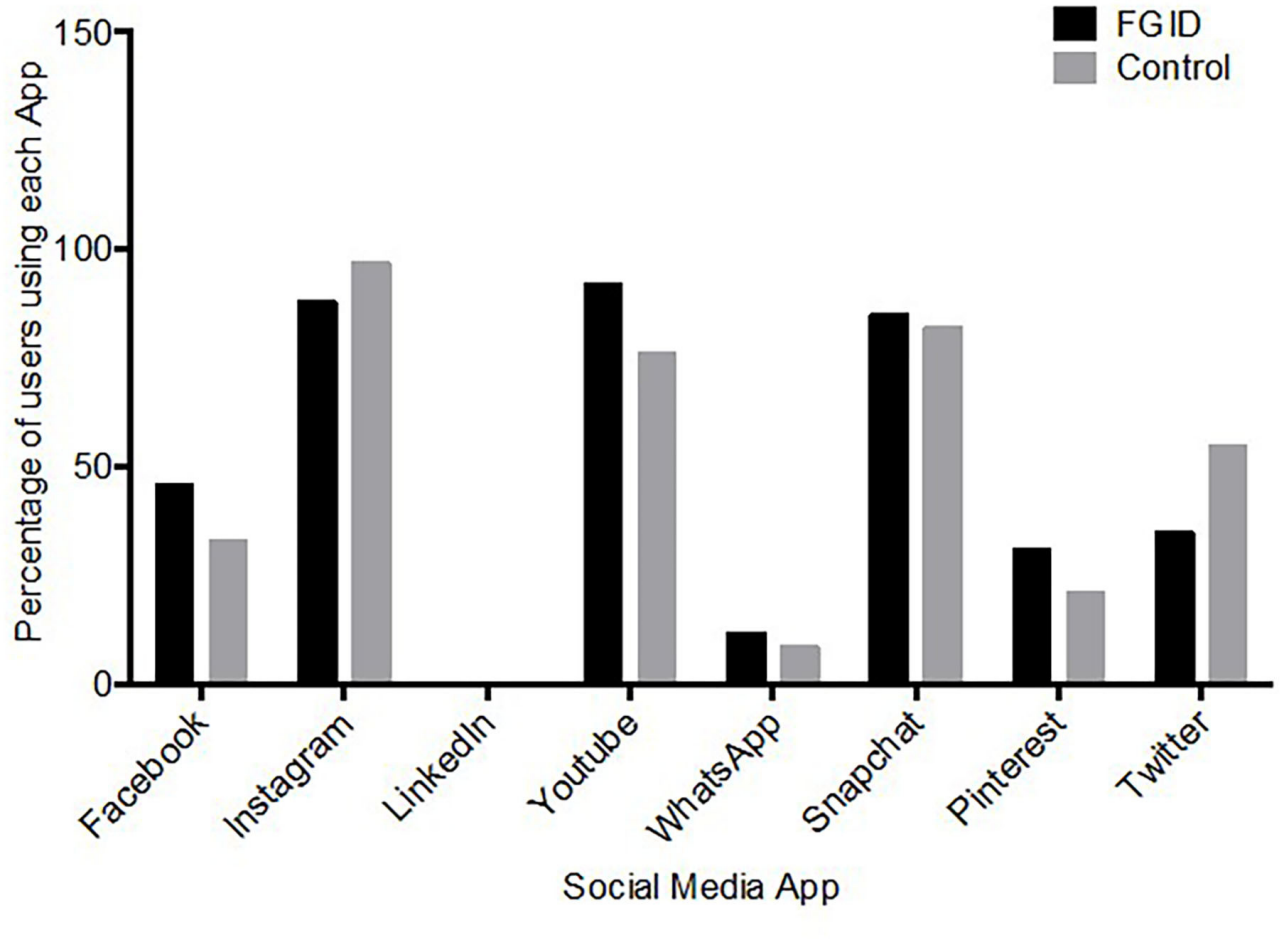
Social media app use, plotted against the number of children using the app. YouTube (92%) and Instagram (88%) were the first and second most popular platforms used by the FGID group, compared to Instagram (97%) and Snapchat (82%) in the control group.

All of the subjects reported using at least one social media platform. The most commonly used platforms were YouTube, Instagram and Snapchat in the FGID group (92, 88, and 85%, respectively) and (76, 97, and 82%, respectively) in controls (Table 2, Figure 1). Although the number of respondents reporting usage of specific platforms varied slightly across the two groups, there were no statistically significant differences. The total number of platforms used was also similar between the two groups (median: 4, IQR: 3–5 for both; *p* = 0.772). There was a high degree of variability in the amount of screen time per day for all respondents, with a minimum of 25 min and a maximum of 960 min, which was not statistically different comparing the cases (median: 309; IQR: 219–375) and controls (median: 297; IQR: 206–449) (*p* = 0.897).

**Figure 2:**
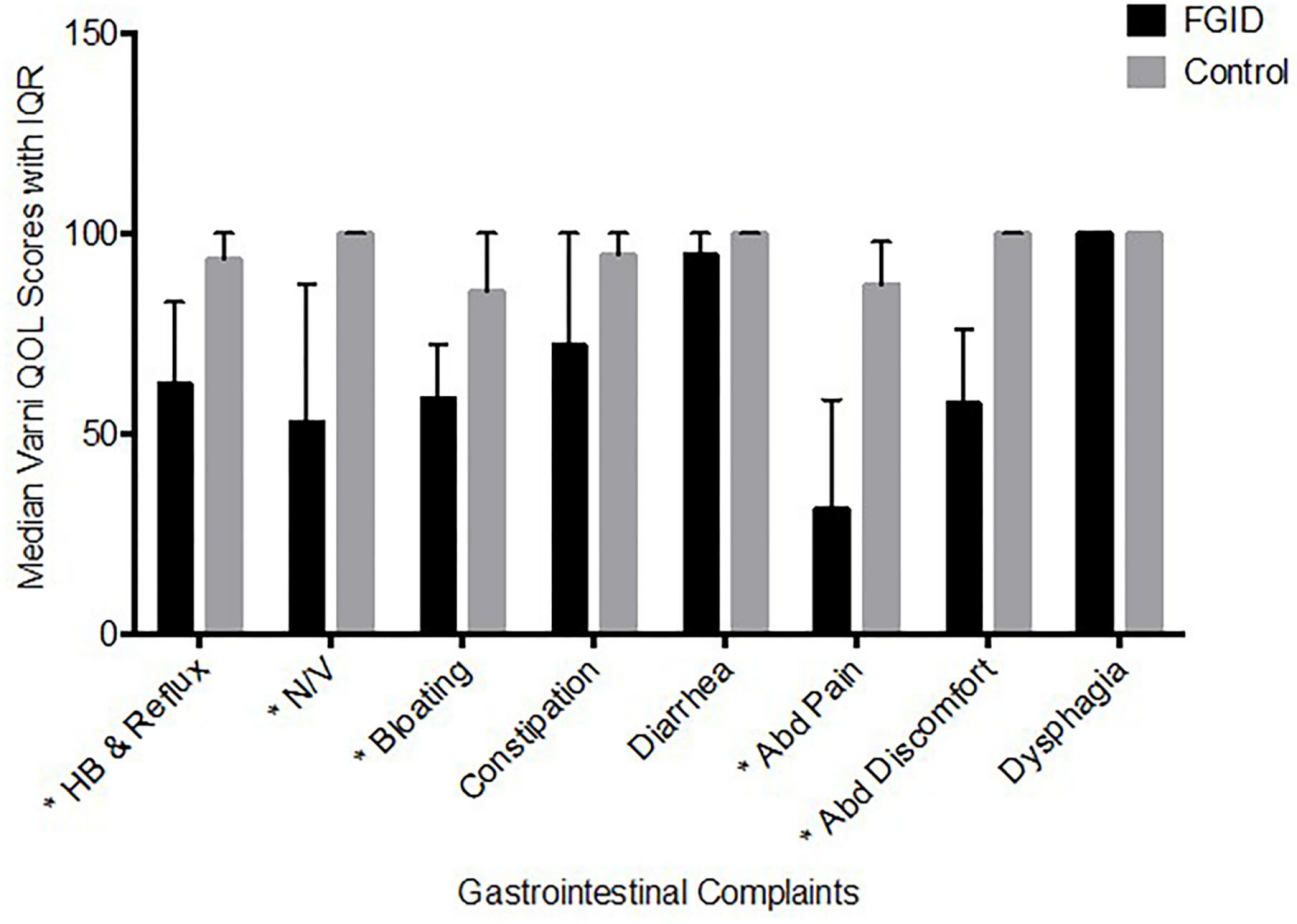
Varni Quality of Life Score based on GI Symptoms (Median ± IQR). The median score across all 8 scales was about a third less for cases (median: 63; IQR: 55–78) than controls (median: 93; IQR: 83–95) (*p* < 0.001). *p < 0.001.

As expected, the cases and controls significantly differed in the PEDS QL scores for six of the eight scales associated with functional GI disorders (Figure 2). The scores were indicative of worse GI-related QL among cases compared to controls for: heartburn and reflux (median: 63 vs. 94), nausea and vomiting (median: 53 vs. 100), gas and bloating (median: 59 vs. 86), stomach pain and hurt (median: 31 vs. 88), and stomach discomfort when eating (median: 58 vs. 100) (*p* < 0.001 for all). The magnitude of difference between cases and controls was smaller for constipation (median: 72 vs. 95; *p* = 0.046), and was not significantly different for diarrhea (median: 95 vs. 100; *p* = 0.083) and trouble swallowing (median: 100 vs. 100; *p* = 0.283). The median score across all 8 scales was ~ a third less for cases (median: 63; IQR: 55–78) than controls (median: 93; IQR: 83–95) (*p* < 0.001).

None of the control patients reported using any medications associated with functional GI disorders. In comparison, only a third of the cases (*n* = 9; 35%) reported not using any medication (*p* < 0.001). Approximately half of the cases (*n* = 12, 46%) reported using one medication, while 4 (15%) and 1 (4%) of the patients reported using two and three medications, respectively. The most commonly reported medication used was hyoscyamine (*n* = 7, 27%), with omeprazole, dicyclomine, and amitriptyline (*n* = 3, 12% for each) being the next most common medications used by the cases.

## Discussion

To our knowledge, this is the first-ever study to look for an association between social media use and functional gastrointestinal disorder in children. In this study, we hypothesized that there is a high prevalence of social media use in adolescents aged 13–18 years with functional gastrointestinal disorders, which is likely correlated with the severity of their symptoms. We hypothesized this because multiple studies have shown a correlation between excessive social media use and psychological issues like depression and anxiety (1–3, 8–10). In a meta-analysis, Hussain and Griffiths (2) showed that the use of social networking websites in adolescents is associated with depression and anxiety. In our literature search, multiple studies showed a higher incidence of depression and anxiety in patients with abdominal pain (7, 11, 12).

One of the interesting studies by Ayonrinde et al. (11) (also called as “Raine Study”) reported a higher incidence of depression, anxiety, being bullied at school, and poor health in adolescents with abdominal pain. von Gontrad et al. (7) and Yacob et al. (12) showed similar findings and reported that children with functional gastrointestinal disorders have significantly higher symptoms of anxiety and depression.

One possible reason for a correlation between social media and depression/anxiety is that social media can affect the gastrointestinal symptoms. Dibb (1) illustrated how social media websites like Facebook can affect an individual in both negative and positive ways and how the overall perception of well-being can be affected by comparing with others online. “Active social media usage refers to online behaviors that facilitate direct exchanges among users” which includes commenting, liking, and messaging. “Passive use is defined as the monitoring of others without direct engagement” (13). Use of social media and correlation with anxiety and depression had been studied by Thorisdottir et al. (14) in Icelandic adolescents. This group showed a higher risk of anxiety and depression with passive social media use. Social media use can affect overall self-esteem including sleep quality, which can affect overall health (15).

Our study did not reveal any significant association between screen time and functional gastrointestinal disorders, as we saw similar results in both the FGID and control group. However, our study had certain limitations, for instance, only patients with iPhones were included (other smartphones and tablets were excluded). Recall bias though limited, still had a role, as part of the study involved a self-reported questionnaire and patients may have been biased in recalling GI symptoms when completing Varni QOL scores. Also, we did not differentiate between active and passive users of social media. Future studies with a more detailed survey on the active and passive use of social media is required. Of note, we did a subgroup analysis, which showed excessive use of screen time in the entertainment, reading, and productivity categories in the functional gastrointestinal disorder group vs. control group (*P* < 0.05). Entertainment apps included gaming apps, music apps, reading apps included kindle, iBooks, google books, etc. Productivity apps included checking email, dropbox, adobe cloud, to name a few. There is a need for further studies to explore this relationship.

Study strengths include clear objectives with primary and secondary aims, design of the study, which included healthy controls to compare with subjects with FGID. Secondly, we used a standardized, validated questionnaire to collect information on the severity of gastrointestinal symptoms and lastly, we used the objective iPhone screen time data to quantify the screen time and avoid recall bias.

## Conclusion

We conclude that the amount of screen time/day and the number of social media platforms used in the last year do not correlate with the severity of abdominal pain in adolescents with functional abdominal pain disorders.

## Data Availability Statement

The raw data supporting the conclusions of this article will be made available by the authors, without undue reservation.

## Ethics Statement

The studies involving human participants were reviewed and approved by University of Texas, Health Science Center, Houston, TX. Written informed consent from the participants' legal guardian/next of kin was not required to participate in this study in accordance with the national legislation and the institutional requirements.

## Author Contributions

ES, SL, SH, and FN were involved in design, methodology, investigation, funding acquisition, analysis, and recruitment of subjects. All authors contributed to the article and approved the submitted version.

## Conflict of Interest

The authors declare that the research was conducted in the absence of any commercial or financial relationships that could be construed as a potential conflict of interest.

## Abbreviations

FGID: Functional Gastrointestinal Disorders
IBS: Irritable Bowel Syndrome
RAP: Recurrent Abdominal Pain
IQR: Inter Quartile Range.

## References

[1] Dibb B. Social media use and perceptions of physical health. Heliyon. (2019) 5:e00989. 10.1016/j.heliyon.2018.e00989 30671555 PMC6327064

[2] Hussain Z Griffiths M. Problematic social networking site use and comorbid psychiatric disorders: a systematic review of recent large-scale studies. Front Psychiatry. (2018) 9:686. 10.3389/fpsyt.2018.00686 30618866 PMC6302102

[3] Primack B Shensa A Escobar-Viera C Barrett E Sidani J Colditz J Use of multiple social media platforms and symptoms of depression and anxiety: a nationally-representative study among U.S. young adults. Comput Hum Behav. (2017) 69:1–9. 10.1016/j.chb.2016.11.013 PMC13268370 42305949

[4] Varni J Bendo C Shulman R Self M Nurko S Franciosi J. Interpretability of the PedsQL gastrointestinal symptoms scales and gastrointestinal worry scales in pediatric patients with functional and organic gastrointestinal diseases. J Pediatr Psychol. (2015) 40:591–601. 10.1093/jpepsy/jsv005 25682210 PMC4469917

[5] Share of people with iPhone in the US 2014-2021 | Statista [WWW Document] Statista. (2020). Available online at: https://www.statista.com/statistics/236550/percentage-of-us-population-that-own-a-iphone-smartphone/ (accessed June 18, 2020).

[6] Lacy BE Patel NK. Rome criteria and a diagnostic approach to irritable bowel syndrome. J Clin Med. (2017) 6:99. 10.3390/jcm6110099 29072609 PMC5704116

[7] von Gontard A Moritz A Thome-Granz S Equit M. Abdominal pain symptoms are associated with anxiety and depression in young children. Acta Paediatr. (2015) 104:1156–63. 10.1111/apa.13134 26194632

[8] Richards D Caldwell P Go H. Impact of social media on the health of children and young people. J Paediatrics Child Health. (2015) 51:1152–7. 10.1111/jpc.13023 26607861

[9] Shensa A Sidani J Dew M Escobar-Viera C Primack B. Social media use and depression and anxiety symptoms: a cluster analysis. Am J Health Behav. (2018) 42:116–28. 10.5993/AJHB.42.2.11 29458520 PMC5904786

[10] Hoge E Bickham D Cantor J. Digital media, anxiety, and depression in children. Pediatrics. (2017) 140:S76–S80. 10.1542/peds.2016-1758G 29093037

[11] Ayonrinde O Ayonrinde O Adams L Sanfilippo F O'Sullivan T Robinson M. The relationship between abdominal pain and emotional wellbeing in children and adolescents in the raine study. Sci Rep. (2020) 10:1646. 10.1038/s41598-020-58543-0 32015372 PMC6997389

[12] Yacob D Di Lorenzo C Bridge J Rosenstein P Onorato M Bravender T Prevalence of pain-predominant functional gastrointestinal disorders and somatic symptoms in patients with anxiety or depressive disorders. J Pediatrics. (2013) 163:767–70. 10.1016/j.jpeds.2013.02.033 23522860

[13] Radovic A Gmelin T Stein B Miller E. Depressed adolescents' positive and negative use of social media. J Adolesc. (2017) 55:5–15. 10.1016/j.adolescence.2016.12.002 27997851 PMC5485251

[14] Thorisdottir I Sigurvinsdottir R Asgeirsdottir B Allegrante J Sigfusdottir I. Active and passive social media use and symptoms of anxiety and depressed mood among icelandic adolescents. Cyberpsychol Behav Soc Netw. (2019) 22:535–42. 10.1089/cyber.2019.0079 31361508

[15] Woods H Scott H. Sleepyteens: social media use in adolescence is associated with poor sleep quality, anxiety, depression and low self-esteem. J Adolesc. (2016) 51:41–9. 10.1016/j.adolescence.2016.05.008 27294324

