# Supplementary material for: Social Media Use in Adolescents With Functional Abdominal Pain
Source: Front Pediatr. 2020 Nov 24;8:592972. doi: 10.3389/fped.2020.592972 (PMC7732463; doi:10.3389/fped.2020.592972)
Supplement: Supplementary file 1 [file Data_Sheet_1.PDF]

a)

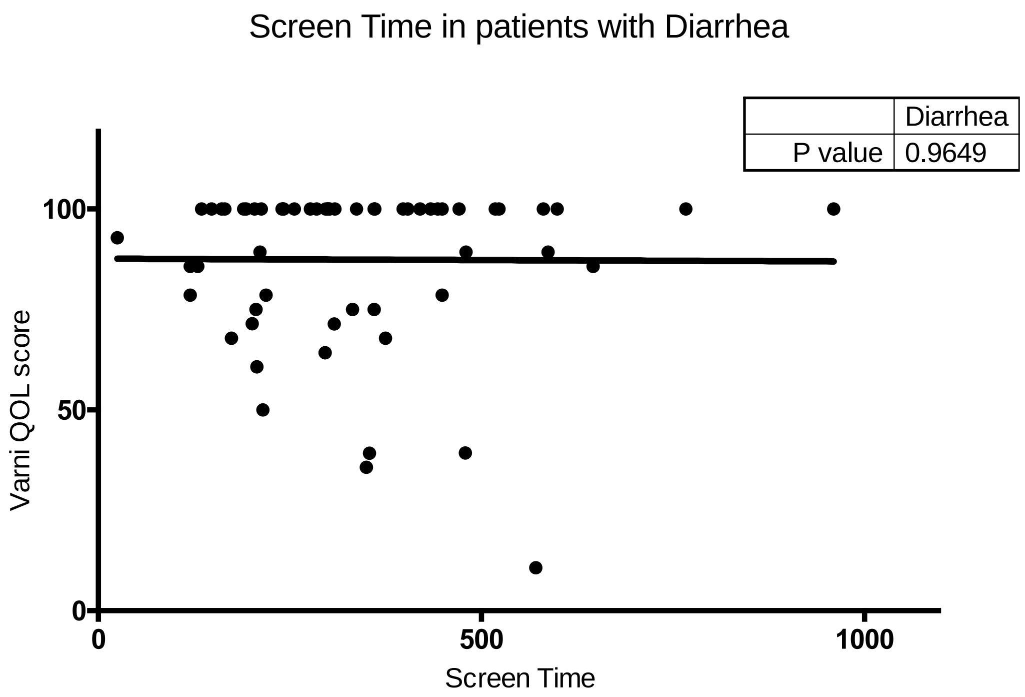
Screen Time in patients with Diarrhea
Diarrhea
P value
0.9649
100
50
0
0
500
1000
Screen Time
Varni QOL score

b)

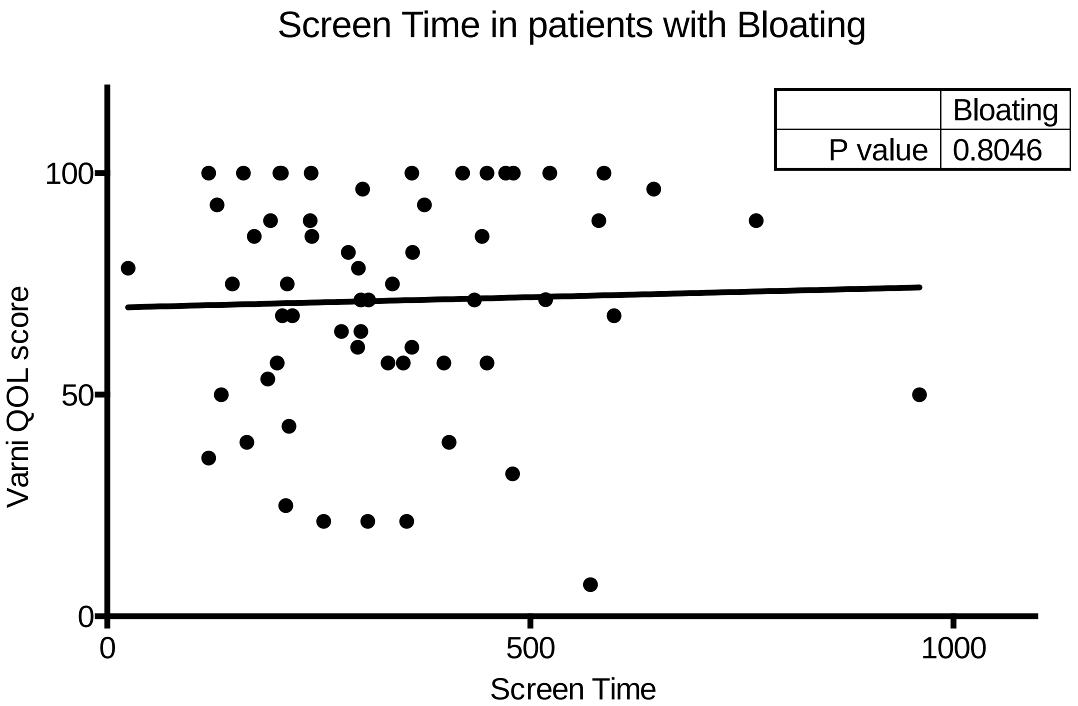
Screen Time in patients with Bloating
Bloating
P value
0.8046
100
50
0
0
500
1000
Screen Time
Varni QOL score

c)

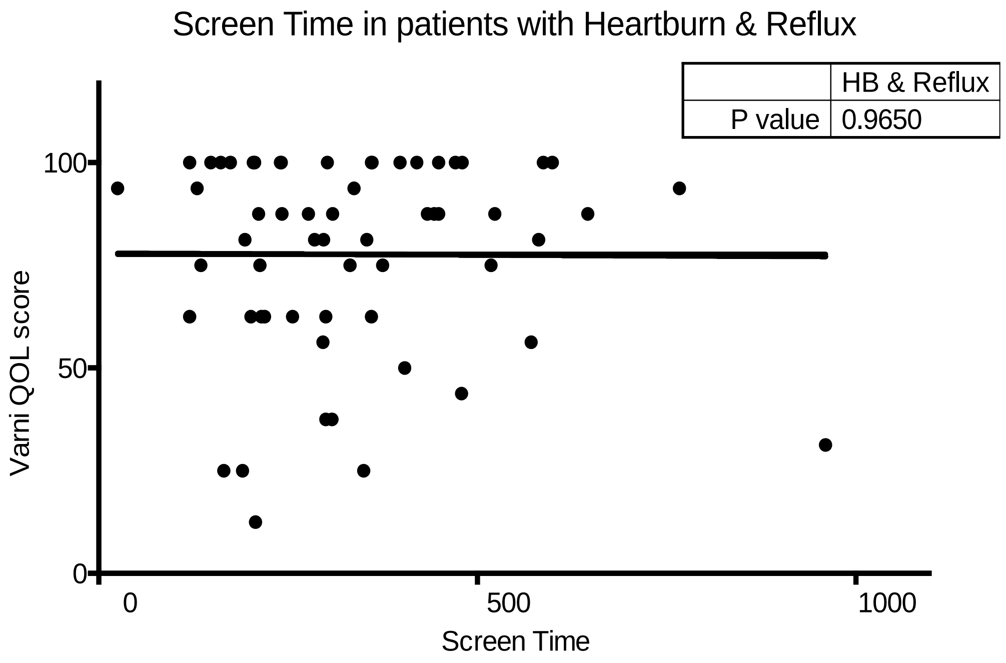
Screen Time in patients with Heartburn & Reflux
HB & Reflux
P value
0.9650
Varni QOL score
100
50
0
0
500
1000
Screen Time

d)

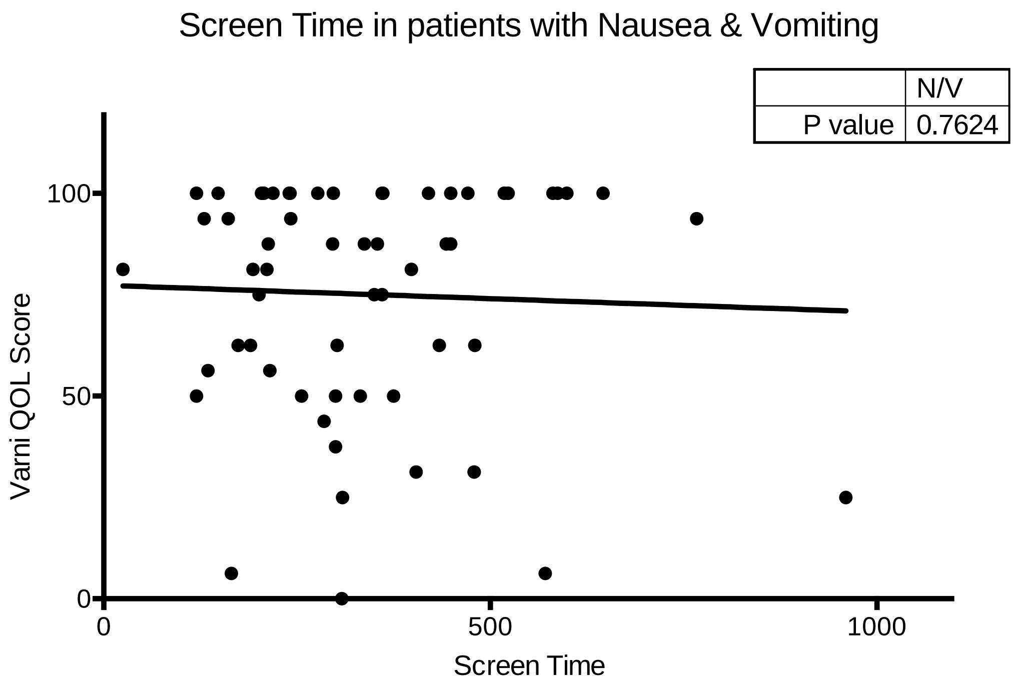
Screen Time in patients with Nausea & Vomiting
N/V
P value
0.7624
Varni QOL Score
100
50
0
0
500
1000
Screen Time

d)

e)

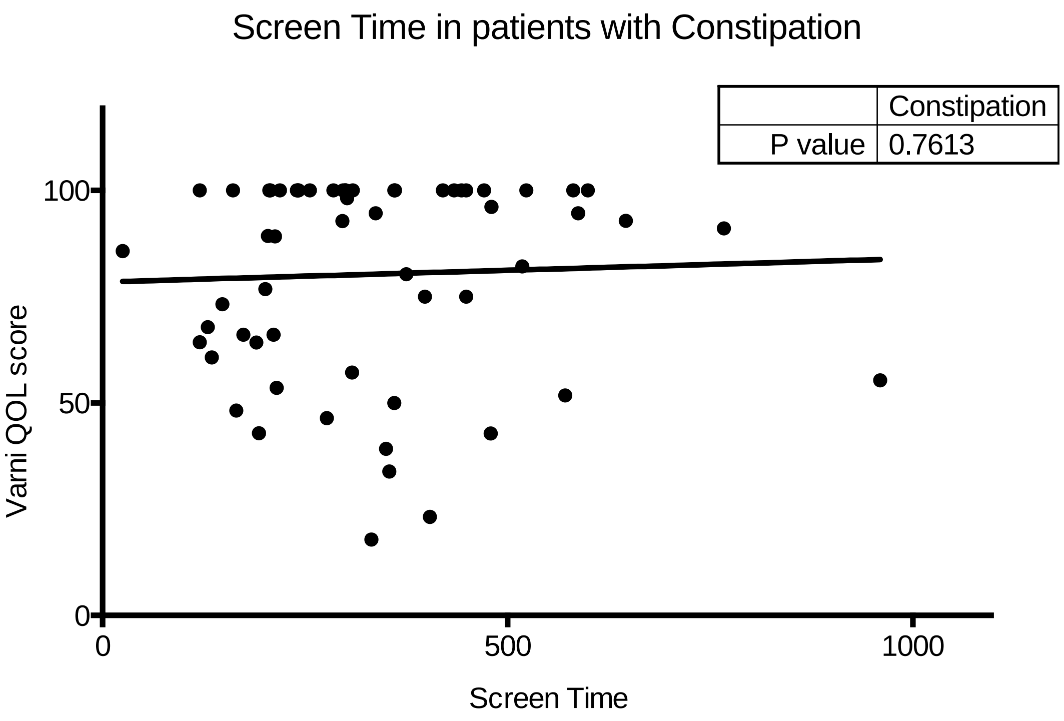

Supplementary Graph 1: No linear correlation found between Screen Time and a) Diarrhea b) Bloating c) Heartburn and reflux d) Nausea and vomiting e) Constipation.
